# Self-Assembly, Antimicrobial Properties and Biodegradability of Ester-Functionalized Choline-Based Surface-Active Ionic Liquids

**DOI:** 10.3390/molecules30061280

**Published:** 2025-03-12

**Authors:** María Teresa García, Elena Bautista, Lourdes Pérez, Sergio Vázquez

**Affiliations:** Department of Surfactants and Nanobiotechnology, Institute for Advanced Chemistry of Catalonia (IQAC-CSIC), 08034 Barcelona, Spain; elena.bautista@iqac.csic.es (E.B.); lourdes.perez@iqac.csic.es (L.P.); sergio.vazquez@iqac.csic.es (S.V.)

**Keywords:** ester-functionalized choline-based ionic liquids, surface activity, antimicrobial properties, hemolytic activity, biodegradability, ecotoxicity

## Abstract

Choline-based ionic liquids (ILs) have gained attention as antimicrobial and antibiofilm agents due to their biocompatibility and tuneable antimicrobial properties. However, a significant drawback of amphiphilic choline-based ILs is their decreasing biodegradability as the alkyl chain length increases. To address this issue and enhance the ecotoxicological profile of these compounds, a labile ester functionality was incorporated into the alkyl side chain. This strategic modification aims to improve biodegradation rates while maintaining the desirable antimicrobial properties of the ILs. A series of ester-functionalized choline-based ionic liquids (CnECholBr) with alkyl chains containing from 10 to 14 carbon atoms were synthesized, and their self-aggregation behaviour in aqueous solutions was studied. Their antimicrobial properties were then tested against clinically significant bacteria and yeasts, as well as their effectiveness in eliminating MRSA and *C. albicans* biofilms. Furthermore, the ecotoxicological properties of these compounds were investigated by assessing their aerobic biodegradability and aquatic toxicity using luminescent bacteria. The results indicated that CnECholBr exhibit higher surface activity and biodegradation rates than non-functionalized choline-based ILs. Conversely, their antimicrobial and antibiofilm activity was found to be lower to that of non-functionalized choline-based ILs. Among the compounds evaluated, the C_12_ECholBr was identified as the most effective antimicrobial and antibiofilm agent.

## 1. Introduction

Antimicrobial resistance (AMR) represents a global threat to public health systems [1]. Misuse and overuse of existing antimicrobials and their dumping or leaking to the general environment, linked to their poor biodegradation, have favored the development and proliferation of AMR [2]. Among the various contributing factors, biofilm development plays a pivotal role [3]. Biofilms are defined as complex communities of microorganisms that adhere to surfaces and are encased within a self-produced extracellular matrix [3,4]. These microbial colonies flourish in diverse environments, including natural, industrial, and clinical settings. Biofilms pose significant challenges across various sectors, particularly in the food industry, public health, and industrial operations [5,6,7]. Effective biofilm control requires the selection of antimicrobials that possess strong antimicrobial properties and the capacity to penetrate the biofilm structure [8].

Ionic liquids (ILs) have gained significant attention for their antimicrobial and antibiofilm properties [4,8,9,10]. The amphiphilic nature of certain ILs enables them to penetrate the extracellular polymeric matrix, enhancing their effectiveness against resilient microbial communities. Furthermore, the tuneable properties of ILs allow for their optimization for specific applications, rendering them promising candidates in antimicrobial strategies across various sectors [11]. However, conventional ILs [12,13], such as those based on imidazolium or phosphonium salts, are poorly biodegradable [14,15,16,17]. This limited biodegradability raises environmental concerns, as these compounds can accumulate and potentially contribute to antimicrobial resistance, a phenomenon documented for quaternary ammonium compounds [18,19]. Consequently, the development of effective yet biodegradable antimicrobial agents is crucial for addressing AMR.

In the search for safer and more sustainable antimicrobial compounds, bio-based ILs have emerged as a promising alternative to environmentally problematic imidazolium-based ILs. Among them, choline-based ILs have attracted considerable attention as antimicrobial and antibiofilm agents due to their biocompatibility and tuneable antimicrobial properties [8,20,21,22,23]. Derived from the essential nutrient choline, these ILs exhibit low toxicity in comparison to traditional ILs, making them suitable for sensitive applications in healthcare and the food industry [20,23]. Choline-based ILs have been demonstrated to be effective in disrupting microbial cell membranes and penetrating biofilms, effectively targeting both planktonic and biofilm-associated microbes [21].

Despite their excellent broad-spectrum antimicrobial properties, amphiphilic choline-based ILs exhibit decreasing biodegradability with increasing alkyl chain length. Only homologues with less than 14 carbon atoms in the hydrocarbon chain are classified as readily biodegradable [21]. The aim of this study was to improve the ecotoxicological profile of these compounds by incorporating a hydrolysis sensitive ester functionality into the alkyl side chain to enhance biodegradation rates. The introduction of a labile bond between the polar head group and the hydrophobic tail was evaluated for its effect on surface activity, antimicrobial and antibiofilm properties, and biodegradation. The results revealed that these new cleavable choline-based ILs exhibited higher surface activity and improved biodegradation rates compared to non-functionalized ILs. However, the modifications also resulted in reduced antimicrobial and antibiofilm activity compared to non-functionalized ILs. Among the newly synthesized compounds, the ionic liquid with a 12-carbon alkyl chain exhibited the highest antimicrobial and antibiofilm activity. This study highlights the trade-offs between improved biodegradability and reduced antimicrobial efficacy in the design of more environmentally friendly ILs.

## 2. Results and Discussion

### 2.1. Synthesis of Ester-Functionalized Choline-Based Ionic Liquids (CnECholBr)

**Figure 1:**
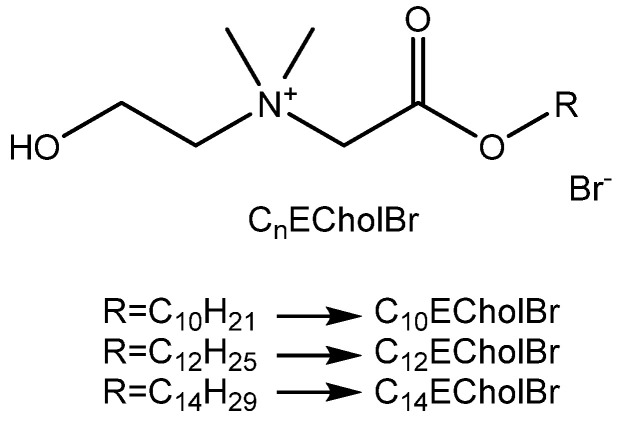
Structure of ester-functionalized choline-based ILs (CnECholBr).

The molecular structure of the ester-functionalized ILs under investigation is shown in Figure 1. The synthesis procedure and characterization data of ester-functionalized cholinium-based ILs, *N*-(2-hydroxyethyl)-*N*,*N*-dimethyl-2-oxo-2-(tetradecyloxy)ethan-1-aminium bromides (CnECholBr), are provided in the Supplementary Materials (Synthesis section, pages S1–S5).

### 2.2. Thermal Stability

The thermal decomposition temperatures of the ester-functionalized cholinium-based ILs have been determined by thermogravimetric analysis (TGA). The characteristic thermal weight loss (TGA) curves for ester-containing cholinium ILs in a nitrogen atmosphere are provided in the Supplementary Materials (Figures S7–S9). A similar thermal decomposition profile was observed for all the ester-functionalized cholinium-based ILs investigated. Two onset decomposition temperatures were identified: the first, occurring at approximately 156–164 °C, resulted in partial oxidation of the IL, while the second, occurring at around 230–236 °C, led to total decomposition of the compound. Table 1 illustrates the onset decomposition temperatures of the investigated ILs in comparison to those of non-functionalized cholinium-based ILs and ester-functionalized and non-functionalized imidazolium and pyridinium based ILs.

It can be observed that the introduction of ester functionality in the alkyl chain has a marked effect on the thermal stability of the cholinium-based ILs, rendering them less stable than the non-functionalized cholinium-based ILs. Similarly, the introduction of ester functionality in the alkyl chain of imidazolium and pyridinium-based ILs results in a notable reduction in the thermal stability of the ILs, in comparison to the corresponding non-functionalized ILs. The onset decomposition temperatures of ester functionalized ILs decrease by approximately 70–80 °C for all the families of ILs, including cholinium, imidazolium, and pyridinium. A comparison of the thermal stability of ILs with different polar head groups reveals that ester-functionalized cholinium-based ILs exhibit a similar thermal stability to ester-functionalized pyridinium salts and a lower stability than ester-functionalized imidazolium salts. Consequently, the operating range is somewhat lower for the former two than for imidazolium-based salts.

### 2.3. Self-Aggregation in Aqueous Solution

The critical micelle concentration (CMC) of the ester-functionalized choline-based ILs in aqueous media was determined by conductivity and fluorescence measurements. The data obtained are presented in Table 2 for subsequent discussion.

#### 2.3.1. Conductivity Measurements

**Figure 2:**
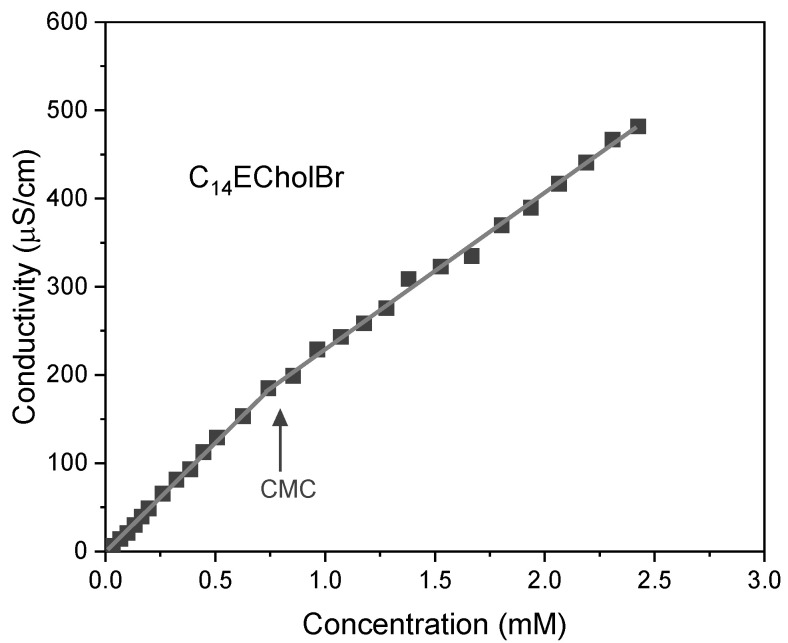
Specific conductivity versus IL concentration in water at 25 °C for C_14_ECholBr.

The change in specific conductivity of ester-functionalized choline-based ILs as a function of their concentration was investigated. Figure 2 shows the conductometric profile of the C_14_ECholBr homologue. Conductometric plots of the other homologues are presented in the Supplemetary Materials (Figures S10 and S11).

In the pre- and postmicellization zones, the specific conductivity values are represented by two straight lines with different slopes. As a consequence of the micelle’s reduced mobility in comparison to the monomers and the binding of certain counterions to it, a pronounced change in slope is observed [6]. The critical micelle concentration (CMC) value was calculated from the point of inflection in the curve. As the length of the alkyl chain increases, the CMC values of ester-functionalized cholinium-based ILs exhibit a progressive decrease (Table 2), a trend observed in non-functionalized cholinium-based ILs as well as for amphiphilic non-functionalized and functionalized imidazolium and pyridinium-based ILs [9,21,24,25,26]. In accordance with the empirical Stauff–Klevens Equation (1) [27], a linear relationship is observed between log CMC and alkyl chain length:

log *cmc* = *A* − *B* × *n* (1)

The values of *A* and *B* are dependent on the characteristics of the polar head group and the impact of each additional methylene group on the critical micelle concentration (CMC), respectively, for a specific homologous series [27]. In the case of ester-functionalized cholinium-based ILs, the values of *A* and *B* were determined to be 4.09 and 0.30, respectively. The ILs in question exhibit the characteristic slope values (0.28–0.30) that have been reported for conventional ionic surfactants [27] and surface-active ILs [9,21,24,25,26].

It was observed that cholinium-based ILs with an ester moiety in the alkyl chain exhibited lower CMC values (Table 2) in comparison to the corresponding non-functionalized homologues [21]. Consequently, ester-functionalized cholinium ILs exhibit a three- to four-fold reduction in CMC values in comparison to their simple alkyl-chain-containing counterparts. The incorporation of an ester functional group in the hydrophobic chain, in proximity to the polar headgroup, appears to result in a decrease in CMC values, which can be attributed to the enhanced H-bonding in the headgroup region [24]. A comparison of the ester-functionalized cholinium-based ILs, CnECholBr (Table 2), with the ester-functionalized imidazolium- and pyridinium-based ILs, CnEMeImBr and CnEPyrBr [24], shows that similar CMC values are obtained for homologues of the different families with the same alkyl chain length. This suggests that all these polar head groups have a similar hydrophobic character.

#### 2.3.2. Fluorescence Measurements

**Figure 3:**
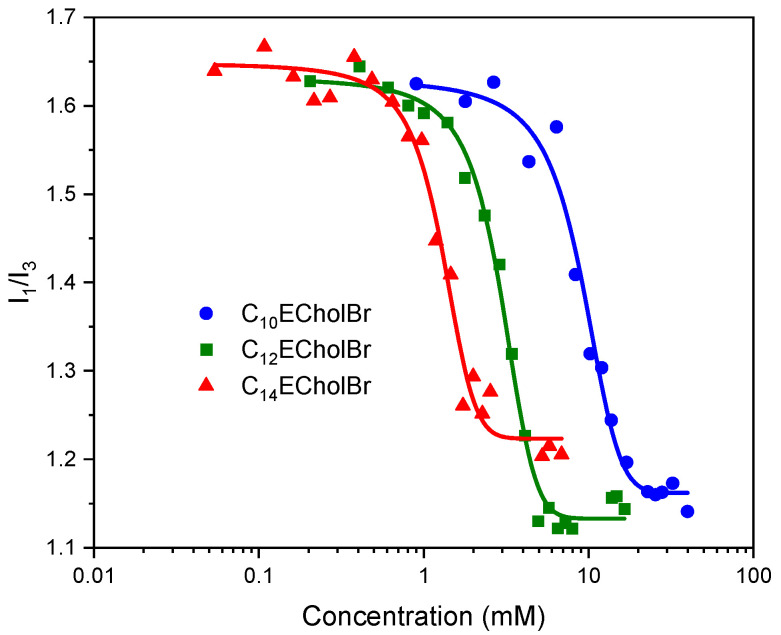
Change in the intensity ratio I_1_/I_3_ of pyrene vs. CnECholBr concentration in water at 25 °C.

The aggregation behavior of ester-functionalized choline-based ILs was investigated through fluorescence measurements using pyrene as a solvatochromic probe. By monitoring the polarity of pyrene’s microenvironment at varying IL concentrations, the intensity ratio of pyrene’s first (I_1_) and third (I_3_) vibronic peaks was analyzed (Figure 3). A sharp decline in the I_1_/I_3_ ratio indicated the formation of IL aggregates. The critical micelle concentration (CMC) was determined as the midpoint of the transition, with the data fitting well to sigmoidal Boltzmann-type curves.

The CMC values obtained from the fluorescence measurements are consistent with those obtained from the conductivity measurements (Table 2), supporting the observed decrease in CMC values with increasing hydrophobicity of the IL.

### 2.4. Antimicrobial and Antibiofilm Properties

In the ongoing search for safe and effective antimicrobial agents, considerable interest has been directed towards the development of bio-based ILs to replace environmentally questionable imidazolium-based ILs. In this context, choline-based ILs are emerging as a novel class of biodegradable antimicrobial agents, displaying a broad spectrum of antibacterial activity. In an effort to enhance the ecotoxicological profile of these compounds, we have introduced an ester functionality in the alkyl side chain to improve their ecological properties, and we have evaluated the effect of such functionalization on the antimicrobial activity of these compounds.

#### 2.4.1. Antimicrobial Activity

The antimicrobial activity of cholinium-based ILs with an ester group in the alkyl chain was evaluated against a range of representative bacteria and fungi. This was achieved by determining their minimum inhibitory concentration (MIC), defined as the concentration required to completely inhibit microbial growth. The concentrations tested ranged from 0 to 256 mg/L. The MIC values obtained for the different alkyl chain homologues of CnECholBr are shown in Table 3.

The MIC values obtained indicate that the antimicrobial activity of cholinium-based ILs with an ester group is contingent on the length of the alkyl chain, a phenomenon that has also been observed in other ILs [9,21,24,25,26]. The antimicrobial activity of ester-functionalized choline-based ILs was found to range from low to moderate against bacteria and yeasts. The results showed that CnECholBr exhibited low toxicity against Gram-negative bacteria, with MIC values above 256 mg/L. The antimicrobial activity of these compounds against some strains of Gram-positive bacteria was moderate and dependent on the length of the alkyl chain, with the most pornounced antimicrobial activity observed against cocci species for chain lengths of 12 or more carbon atoms. Among the homologues examined, C_12_ECholBr was identified as the most potent antimicrobial agent against bacteria. No antimicrobial activity was observed for the C10 and C14 homologues against fungal strains; however, moderate activity of C_12_ECholBr was identified against *Candida albicans* and *Candida glabrata*.

Compared to non-functionalized cholinium-based ILs [21], the incorporation of an ester group within the alkyl side chain close to the cationic headgroup results in a significant reduction in the antimicrobial activity (Table 3). This behaviour is contrary to that of ester-functionalized imidazolium and pyridinium ILs, which have been shown to be more effective antimicrobial agents than their non-functionalized counterparts [9,24]. However, as with the other families of ester-functionalized ILs mentioned above, ester-functionalized choline-based ILs with an alkyl chain length of 12 carbon atoms demonstrated the highest efficiency as antimicrobial agent. It is interesting to note that, despite the incorporation of ester functionality, resulting in an increase in surface activity, there has been no observed increase in antimicrobial activity. This could be explained by the reduced stability of these compounds in water due to their susceptibility to chemical hydrolysis.

#### 2.4.2. Antibiofilm Activity

**Figure 4:**
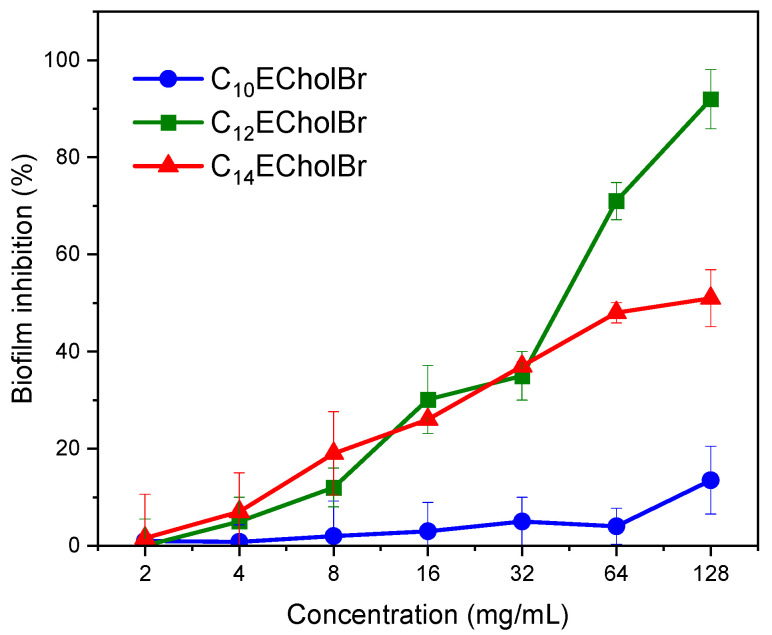
Effectiveness of CnECholBr in inhibiting MRSA biofilm formation. The percentage of biofilm inhibition was calculated by comparing treated samples to two reference points: untreated bacteria (set as 100%) and sterile control wells (set as 0%). Each data point shown represents the average of three separate experimental replicates.

To assess the ability of ester-functionalized cholinium-based ILs to suppress MRSA biofilm formation, CnECholBr samples were introduced to newly prepared bacterial suspensions. These mixtures were then incubated for 24 h at 37 °C. Subsequently, crystal violet (CV) staining was employed to quantify the biofilm biomass. The findings from this experiment are illustrated in Figure 4.

Among the C_n_CholBr homologues examined, C_12_ECholBr was found to significantly inhibit biofilm formation at concentrations above 64 µg/mL, reaching an inhibition rate of more than 90% at 128 µg/mL. In contrast, the C14 homologue showed no more than 50% inhibition at the highest concentration tested (128 µg/mL), while C10 showed less than 15% inhibition at the highest concentration tested (128 µg/mL). In comparison to the non-functionalized choline-derived ILs, their capacity to inhibit MRSA bacterial biofilm is clearly inferior, as the former demonstrated biofilm inhibition values close to 100% at lower IL concentrations [21].

**Figure 5:**
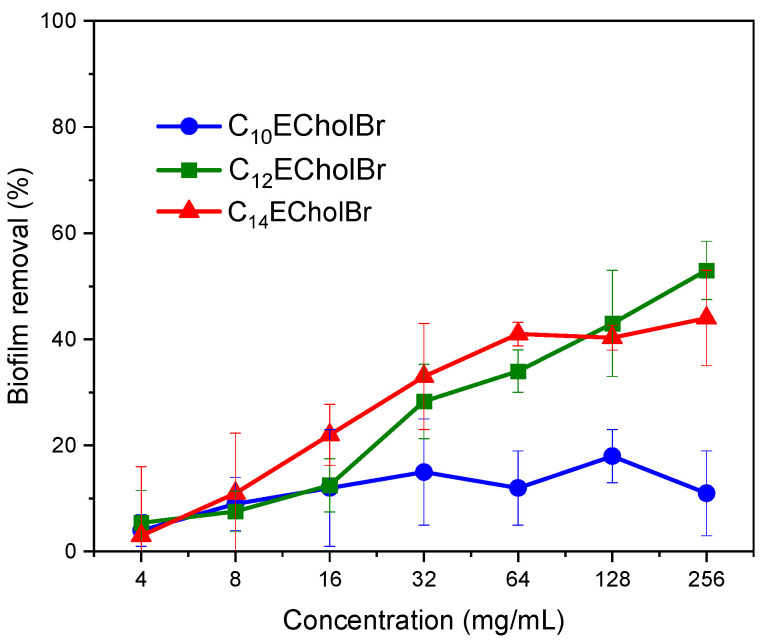
Results of the biofilm eradication tests of ester-functionalized choline-based ILs against MRSA.

The capacity of the ester-functionalised cholinium-based ILs to eradicate established MRSA biofilms was evaluated through the performance of eradication assays on microtiter plates. CV staining was applied to measure biofilm biomass. The results are shown in Figure 5.

The ester-functionalised choline-based ILs exhibited limited efficacy in dispersing mature MRSA biofilms, as evidenced by CV staining. The percentage of biofilm biomass removal did not exceed 60% for either C12 or C14 homologues, even at the highest tested concentration of 256 µg/mL. The C12 homologue demonstrated a marginal improvement in biofilm removal compared to its C14 counterpart. Conversely, the C10 homologue exhibited minimal activity, attaining less than 20% biofilm removal. It is evident that the capacity of CnECholBr to remove MRSA biofilm biomass is notably lower than that reported for non-functionalized choline-based ILs [21].

**Figure 6:**
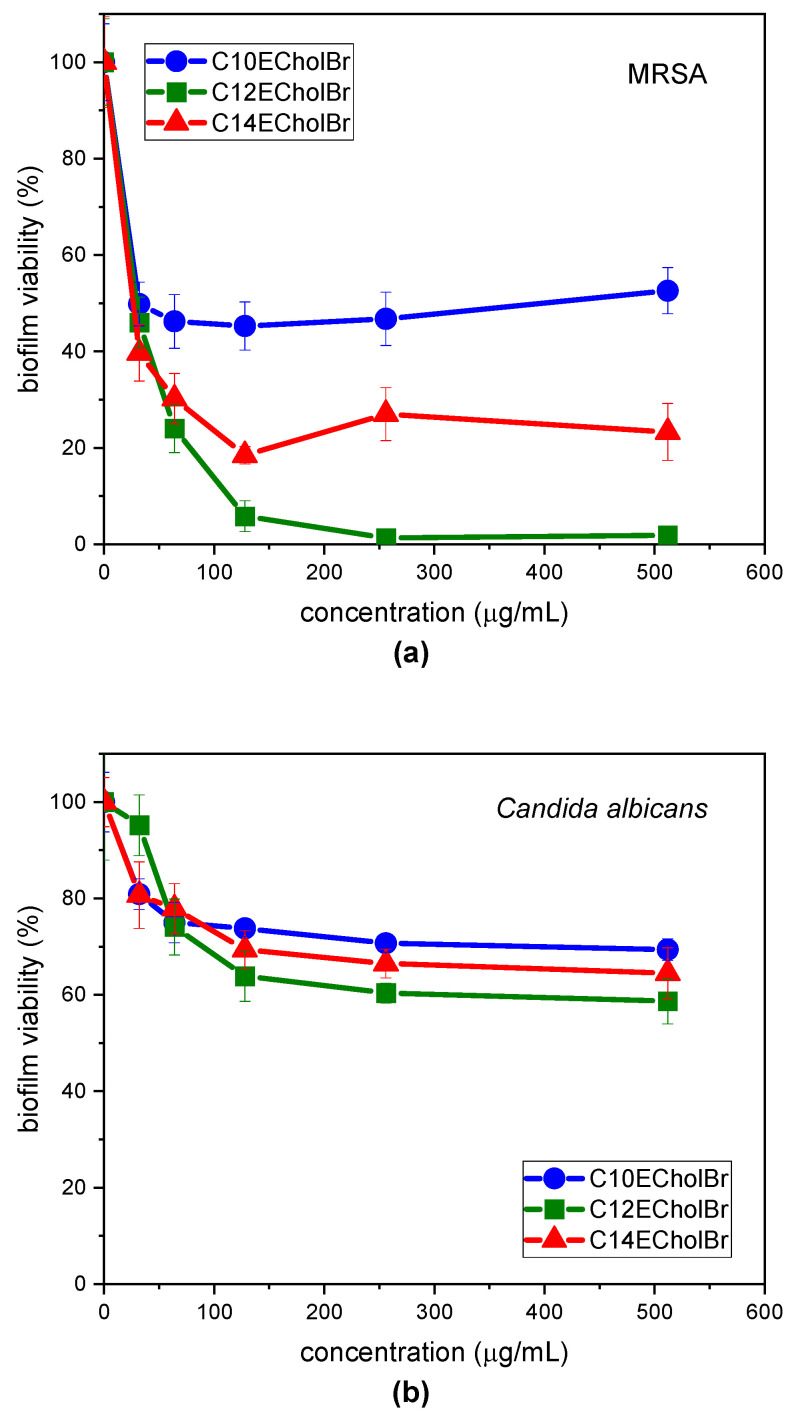
Quantification of viable cells in MRSA (**a**) and *C. albicans* (**b**) biofilms by MTT method. The *y*-axis represents the cell numbers relative to the negative control. Error bars are generated from 6 replicates.

The biocidal activity of the ester-functionalized choline-based ILs on bacterial and fungal cells in biofilms was assessed by MTT cell viability assays. CnECholBr were evaluated for their potential to kill cells in pre-established biofilms of MRSA and *C. albicans*, two clinically relevant pathogens. Pre-formed biofilms on 48-wells plates were treated with increasing concentrations of the ester-functionalized choline-based ILs and the cell viability of the sessile cells was quantified by the MTT method. The results obtained for MRSA and *C. albicans* biofilms are shown in Figure 6a and Figure 6b, respectively.

The metabolic activity of bacterial cells in MRSA biofilms is reduced when treated with ester-functionalized choline-based ILs (Figure 6a). However, the behaviour of these compounds varies depending on the alkyl chain length of the homologue. For C10E and C14E, a certain degree of inhibition is observed at the lowest concentrations that were examined. For C10ECholBr, a 50% reduction in cell viability is attained at 32 µg/mL but this value does not increase with increasing concentration. For C_14_ECholBr, a dose-response relationship is observed for concentrations ranging from 32 to 128 µg/mL, after which the percentage of cell viability remains constant (around 30% cell viability), although concentration was increased. In contrast to the 10 and 14C homologues, C_12_ECholBr does exhibit a discernible dose-response activity, resulting in a complete inhibition of cell viability (>95% for concentrations ≥ 128 µg/mL). This concentration is equivalent to twofold the concentration required to inhibit growth in planktonic bacteria (Table 3). Consequently, while all three homologues demonstrate a reduction in cell viability, it is noteworthy that only C_12_ECholBr exhibits the capacity to completely eliminate bacterial cells within MRSA biofilms.

When *C. albicans* biofilms were treated with CnECholBr (Figure 6b), it can be seen that the metabolic activity of fungal cells in pre-established biofilms decreased with increasing IL concentration, although it tended to stabilise at CnECholBr concentrations above 128 µg/mL. The reduction in biofilm viability did not exceed 40% for any of the homologues tested at the higher concentration tested. The C_12_ECholBr exhibited a sligtly superior capacity for cell death compared to the other two homologues, with the biocidal activity ranking as C12 > C14 > C10. This finding is consistent with the observed order of antifungal activity of these compounds against planktonic fungal cells (Table 3).

### 2.5. Hemolytic Activity

**Figure 7:**
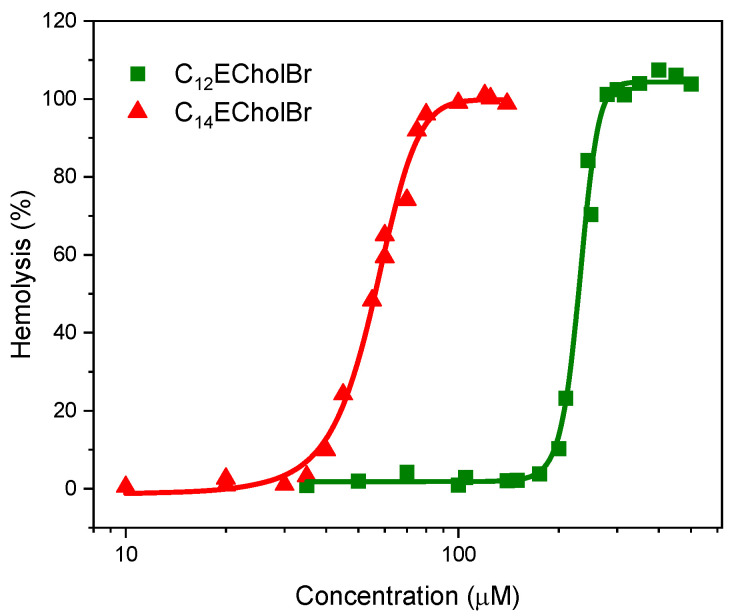
Dose-response curves for the hemolytic activity of CnECholBr.

To perform an initial assessment of cell toxicity, hemolysis has been employed. The hemolytic activity of ester-functionalized choline-based ILs was determined by mesuring the release of haemoglobin in erythrocytes from rabbit blood samples in the presence of these compounds. The HC_50_ values, representing the concentracion inducing 50% hemolysis, were calculated from the plots of the percentages of hemolysis versus CnECholBr concentration (Figure 7). The HC_50_ values are compiled in Table 4. These data provide an indication of the toxicity of these ILs to mammalian cells.

In general, these ILs demonstrate moderate or low hemolytic activity, with HC_50_ values greater than 50 µM. Their hemolytic activity increases significantly with the lengthening of the alkyl chain. For instance, C_10_ECholBr displays negligible hemolytic activity (HC_50_ > 2500 µM), whereas C_14_ECholBr exhibits the higher hemolytic activity of the series, with a HC_50_ value of 56 µM. A comparison of CnECholBr with non-functionalized choline-based ILs shows that they are more hemolytic at the same alkyl chain lengths. This can be attributed to the presence of two additional carbon atoms in the ester group linking the hydrophobic alkyl chain and the polar head group, which increases the hydrophobicity of the molecule. This has previously been reported as one of the main factors governing the hemolytic activity of other surface active molecules [28,29]. It is noteworthy that of the two CnECholBr homologues with antimicrobial activity, C12 and C14, the C12 homologue has the higher antimicrobial activity and the lower hemolytic activity.

### 2.6. Aerobic Biodegradability

To evaluate the impact of incorporting a cleavable group within the side alkyl chain of the cholinium-based ILs on their ecotoxicological profile, the biodegradability of these compounds was investigated. The aerobic biodegradation of ester-functionalized cholinium-based ILs was evaluated using the CO_2_ Headspace Test (OECD 310). This method measured CO_2_ production resulting from the mineralization of the ILs by quantifying the inorganic carbon generated in the test vessels, compared to that produced in blank vessels. Biodegradation levels were expressed as a percentage of the theoretical maximum inorganic carbon formation, calculated based on the initial concentration of ionic liquid. Table 5 presents the mineralization percentages for these ILs, along with the 28-day biodegradation average and the corresponding 95% confidence interval.

**Figure 8:**
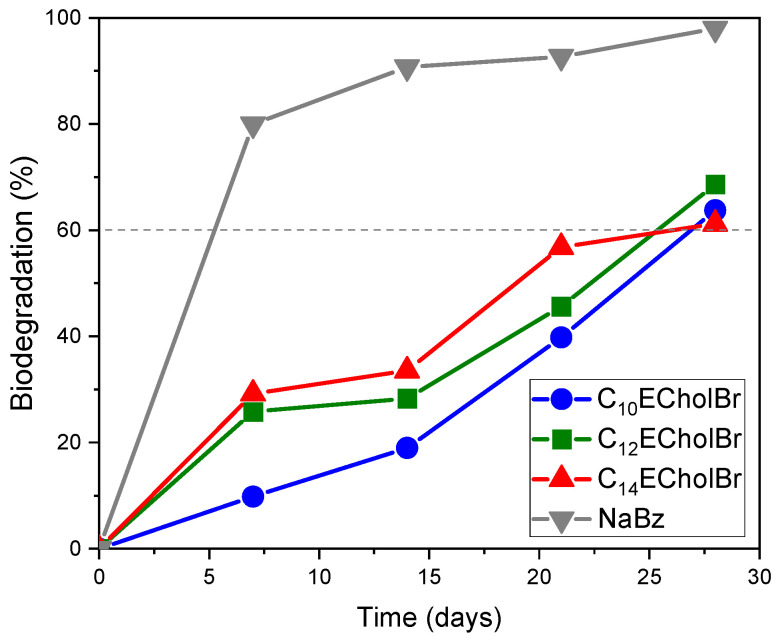
Biodegradation curves of CnECholBr and reference substance NaBz by the CO_2_ headspace test. Dotted line indicates the pass level for ready biodegradability (60% of theoretical CO_2_ production).

Ester-functionalized choline-based ILs showed a significant degree of mineralization under the experimental conditions over the 28-day period (61 to 69%), with all of them attaining the level of biodegradation required to be considered as readily biodegradable compounds, i.e., greater than 60% (Figure 8).

In comparison with non-functionalized cholinium based ILs, CnECholBr show faster biodegradation kinetics, likely attributable to the presence of an ester group susceptible to chemical or enzymatic hydrolysis. The C_14_ECholBr homologue can be regarded as readily biodegradable, as it reaches a biodegradation level of more than 60% within 28 days, whereas its corresponding non-functionalized homologue, C_14_CholBr, requires a longer period of time (42 days) to reach the same threshold. Therefore, the former can be considered as readily biodegradable, while the non-functionalized analogue cannot be considered as such. The incorporation of an ester functionality in the side chain has been demonstrated to enhance the biodegradation kinetics of choline-based surfactants, with the C10–C14 homologues studied being considered as readily biodegradable under aerobic conditions. Overall, the results showed that the incorporation of an ester functionality in the side chain improves the biodegradation kinetics of choline-based surfactants, with the C10–C14 homologues studied being considered as readily biodegradable under aerobic conditions.

### 2.7. Aquatic Toxicity

To assess the aquatic toxicity of ester-functionalized cholinium-based ILs, a series of toxicity tests were conducted using the saltwater bacterium *Vibrio fischeri*. The bioluminescence inhibition assay with *V. fischeri* is a well-standardized and frequently employed method in ecotoxicological studies due to its sensitivity and reliability. The experimental procedure involved exposing *V. fischeri* to increasing concentrations of ILs and measuring the reduction in bioluminescence after a 30-min period. This bioluminescence reduction serves as a quantitative indicator of the ILs’ aquatic toxicity in marine bacteria. The outcomes of these toxicity tests are presented in Table 6.

**Figure 9:**
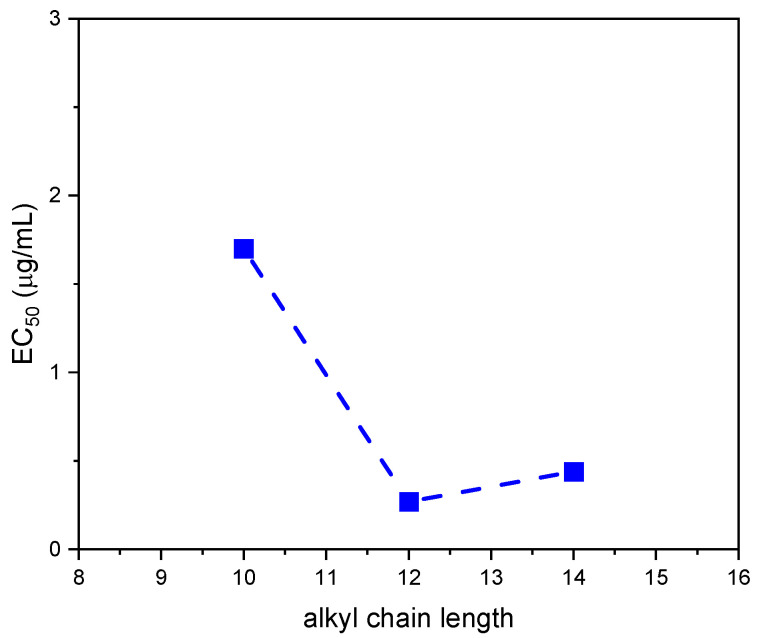
Aquatic toxicity of CnECholBr against luminescent marine bacteria (*Vibrio fisheri*) as a function of the alkyl chain length.

The concentration of IL required to reduce luminescence in bacteria to 50% of its original level (EC_50_) was found to range from 1.7 to 0.27 mg/L. When the toxicity values are plotted against the alkyl chain length of the IL, it is observed that toxicity increases (lower EC_50_ values) with increasing alkyl chain length from 10 to 12 carbon atoms, whereas toxicity is maintained or slightly decreased for the C_14_ECholBr (Figure 9). This suggests that the cation hydrophobicity is a primary factor contributing to the aquatic toxicity of ester-functionalized cholinium-based ILs. The tendency to stabilise or slightly decrease the toxicity of ILs with more than 12 carbon atoms in the alkyl side chain could be attributed to the reduced solubility and consequently reduced bioavailability of C_14_ECholBr in the saline media of the *Vibrio fisheri* bioassay.

Ester-functionalized cholinium-based ILs with long alkyl chains exhibit similar aquatic toxicity to non-functionalized cholinium ILs towards luminescent marine bacteria. This finding reinforces the prevailing idea that there is a strong correlation between toxicity and alkyl side chain length of ILs.

## 3. Materials and Methods

### 3.1. Synthesis of Ester-Functionalised Choline-Based ILs

The synthesis of *N*-(2-hydroxyethyl)-*N*,*N*-dimethyl-2-oxo-2-(tetradecyloxy)ethan-1-aminium bromides(CnECholBr) was performed in two steps according to standard methodology [24,30]. First, commercially available alcohols were reacted with bromoacetyl bromide to obtain the desired alkylating agents. The subsequent alkylation of 2-dimethylaminoethanol with these alkylating agents allows us to obtain the corresponding ester-functionalized cholinum-based bromide. The synthesis procedure and characterisation data of the cholinium-based ILs are given in the Supplementary Materials.

### 3.2. Thermal Stability Measurements

Thermogravimetric analysis was performed using a simultaneous TGA/DSC instrument (model Discovery SDT 650, TA Instruments, New Castle, DE, USA). The instrument was calibrated with indium, zinc, and sapphire. Analyses were performed under nitrogen purge at a Flow rate of 100 mL/min from r.t. to 550 °C at 10 °C/min. Samples were weighed in 90 µL alumina crucibles using a microbalance (model XPR2, Mettler Toledo, Hong Kong). Data were analysed using the TA Instruments TRIOS software version 5.1.

### 3.3. Conductivity Measurements

Conductivity measurements were carried out at 25 °C using an Orion conductivity cell 913005MD with an epoxy/graphite electrode and a Thermo Orion 5 Star multiparameter instrument with a cell constant of 0.475 cm^−1^.

### 3.4. Fluorescence Measurements

A Shimadzu RF 540 spectrofluorometer equipped with cell holder thermostatical controlled at 25 °C was used to detect steady-state fluorescence. The fluorescent probe, pyrene, was used at a concentration of 1 × 10^−6^ M. After irradiation at 332 nm, the fluorescence emission spectra of pyrene dissolved in aqueous CnECholBr solutions were recorded from 340 to 450 nm. The 370–400 nm region of the steady-state fluorescence emission spectrum of pyrede shows a fine structure, and the polarity of the surrounding environment strongly influences the nature and strength of these bands. The ratio of the first to the third vibronic peak, or I_1_/I_3_, which shows the greatest solvent dependence, was used to calculate the critical micelle concentration (CMC) of the ILs in aqueous solution [26].

### 3.5. Antimicrobial Activity

#### 3.5.1. Antibacterial Activity

Antibacterial assays were performed against the following microorganisms: *Bacillus subtilis* ATCC 6633, *Staphylococcus epidermidis* ATCC 12228, *Staphylococcus aureus* ATCC 29213, *Micrococcus luteus* ATCC10240, *Listeria monocytogenes* ATCC 15313, methicillin-resistant *Staphylococcus aureus* ATCC 43300, *Escherichia coli* ATCC 8739, *Acinetobacter baumannii* ATCC 19606, *Klebsiella aerogenes* ATCC 13048, *Pseudomonas aeruginosa* 9027, and *Salmonella enterica* ATCC14208. The antibacterial activity was assessed in vitro using the minimum inhibitory concentration (MIC) method [31]. MIC is defined as the lowest concentration of an antimicrobial agent that prevents visible bacterial growth after 24 h of incubation at 37 °C. The ionic liquids were dissolved in Mueller–Hinton broth (MBH) at concentrations ranging from 1 to 256 μg/mL. The broth was added (200 μL) to appropriate wells of a 96-well polystyrene microtitre plate. Then, 10 μL of a nutrient broth starter culture of each bacterial strain was added to achieve final inocula of approximately 5 × 10^−5^ colony forming units (CFU)/mL. A growth control using nutrient broth medium without the ionic liquid was included. The plates were incubated 37 °C for 24 h. Bacterial growth was initially observed visually, with increased turbidity indicating cell growth [21]. To confirm visual MIC values, 20 μL of 0.015% *w*/*v* of resazurin was added to each well and incubated at 37 °C for approximately 90 min. A color change of resazurine from blue to pink indicated bacterial metabolic activity. All the experiments were performed in triplicate to ensure reproducibility.

#### 3.5.2. Antifungal Activity

The antifungal activity was evaluated against the following yeasts: *Candida albicans* ATCC 90028, *Candida tropicalis* ATCC 7349, *Candida parapsilosis* ATCC 22019, *Candida glabrata* ATCC 66032. Frozen yeast stocks were inoculated into Sabourauds’s agar (SBA) and culture plates and incubated at 35 °C for 24 h. Three to four colonies of each strain were dispersed in sterile medium, Roswell Park Memorial Institute medium (RPMI 1640 medium), to obtain dispersions with a turbidity equivalent to 0.5 on the MacFarland scale (McFarland DEN-1B Grant-bio model densitometer). Minimum fungicidal concentrations (MFC) values—the lowest concentration of an antimicrobial agent that prevents the establishment of visible microbial growth after 24 h at 30 °C—were used to determine in vitro antifungal activity. MFCs were determined using a broth microdilution assay [31]. Serial dilutions of each IL, ranging from 512 to 2 µg/mL, were dissolved in RPMI 1640 medium and dispensed (100 µL) into the appropriate wells of a 96-well polystyrene microtiter plate. The nutrient broth starter culture of each yeast strain (100 μL) was added to achieve a final inoculum of approximately 7.5 × 105 CFU per mL for yeast. Nutrient broth medium without the compound was used as a growth control. The development of turbidity in an inoculated medium is a function of growth and reflects an increase in both mass and cell number. To confirm this observation, 20 μL of resazurin at 0.015% *w*/*v* was added to each well and allowed to react for approximately 2 h at 30 °C. After the incubation period, the indicator of microbial growth, i.e., a change from blue to pink, confirmed the MIC value.

### 3.6. Antibiofilm Activity

Antibiofilm activity was assessed against biofilm-forming strains of methicillin-resistant *Staphylococcus aureus* (MRSA) ATCC 43300 and *Candida albicans* ATCC 90028. The biofilm inhibition and eradication capacities of ILs were evaluated against adherent bacterial biofilms grown in 96-well microtiter plates [32]. Bacteria were grown on Tryptic soy agar at 37 °C for 24 h and the fungi on Sabouraud agar at 30 °C for 24 h. Bacteria were suspended in lysogeny broth containing glucose (1%) at 1.5 × 10^8^ CFU/mL and yeasts were suspended in RPMI medium at 1.5 × 10^7^ CFU/mL.

#### 3.6.1. Biofilm Inhibition Procedure

To assess the effect of ionic liquids on bacterial biofilm formation, we employed a microplate assay. We added 100 μL of IL at varying concentrations (2–128 μg/mL, using two-fold serial dilutions) to each well of a 96-well microplate, followed by 100 μL of diluted bacterial suspension. After incubating the plates at 37 °C for 24 h, we measured bacterial growth by recording the optical density at 600 nm (OD600). We then discarded the spent media, fixed the biofilm with methanol, and stained it with crystal violet. Following a water rinse, we dissolved the stained biofilm using an ethanol-acetic acid solution and measured the absorbance at 570 nm to quantify biofilm formation. To ensure reproducibility, we performed each assay in triplicate and averaged the results.

#### 3.6.2. Biofilm Eradication Procedure

To evaluate the effect of ionic liquids on mature biofilms, we employed a modified microtiter plate assay. We first generated mature biofilms by incubating 200 μL of diluted bacterial suspension in each well of a 96-well plate for 24 h at 37 °C. After gently washing the biofilms with PBS, we added 200 μL of ionic liquid solution at various concentrations (4–256 μg/mL, using two-fold serial dilutions) to each well and incubated for an additional 24 h at 37 °C. Following treatment, we fixed the biofilms with 150 μL methanol for 10 min. The subsequent crystal violet (CV) staining and quantification steps were performed as described in the previous biofilm formation assay (Section 3.6.1), allowing us to assess the impact of ionic liquids on established biofilms.

#### 3.6.3. Assessment of Biofilm Cell Viability

For mature biofilm formation, 200 μL of the bacterial or fungal suspension was added to each well in a 48-well microplate, and incubated for 24 h at 37 °C for bacteria and 30 °C for yeast. The wells were then washed three times with PBS and 200 µL of each test solution with different concentrations of surfactants prepared in MHB (for bacteria) or RPMI medium (for yeasts) was added to each well containing the preformed biofilm. The plates were incubated for 24 h at 30 °C. The medium was removed and the wells were then washed twice with PBS. MTT solution (200 μL) was added to each well and the plate was incubated for 4 h. The MTT was then removed and 200 μL of DMSO was added to solubilize the biofilm. The OD value was recorded at 540 nm. Controls were performed with non-ionic liquids. Each assay was run four times and the results were averaged.

### 3.7. Hemolytic Activity

Rabbit blood samples, used to prepare the erythrocyte suspension, were obtained from the animal facility of the Research and Development Center (CID) of the Spanish National Research Council (CID-CSIC) in Barcelona, Spain. The collection of fresh blood from rabbits adhered strictly to the bioethical guidelines stipulated by Spanish legislation, ensuring ethical treatment of the animals and compliance with research protocols. Heparinized blood samples were centrifuged for 10 min at 3000 rpm. The supernatant containing the white cells was discarded, while the erythrocytes were washed three times in PBS (pH 7.4). Erythrocytes were then suspended in PBS at a cell density of 8 × 10^9^ cells/mL. A series of different volumes of a concentrated IL solution (10 to 80 μL) were placed in Eppendorf tubes containing 25 μL of erythrocyte suspension and PBS was added to each tube to a total volume of 1 mL [33]. Samples were shaken for 10 min at rt and the tubes were centrifuged (5 min at 10,000 rpm). The percentage of hemolysis was calculated by comparing the absorbance (540 nm) of the supernatant of the samples containing surfactants with that of the hemolyzed control with distilled water. The HC_50_ (concentration of surfactant that causes 50% hemolysis) was determined from the concentration-response curves.

### 3.8. Aerobic Biodegradability

The aerobic biodegradability of the ILs was investigated through the application of the OECD 310 method (CO_2_ headspace test) [34]. This standard method assesses the mineralization of an organic compound in an aqueous medium through the measurement of CO_2_ production. The rate of biodegradation of ester-functionalized choline-based ILs was calculated by monitoring the production of CO_2_ as these compounds were mineralized by microorganisms over time. A mixed population of microorganisms derived from activated sludge from a sewage treatment plant (Manresa, Barcelona) was introduced to each IL solution at a concentration of 10 mg C/L, serving as the sole source of carbon and energy. The reference substance was sodium benzoate (NaBz) at a concentration of 20 mg C/L. The biodegradation assays were conducted over a period of 28 days. Inhibition studies using binary mixtures of NaBz and CnECholBr at 20 and 10 mg C/L, respectively, were also carried out for each test IL. Biodegradation was assessed using a carbon analyzer (Shimadzu TOC-VCSH, Shimadzu Corporation, Kyoto, Japan), and assessed as the net increase in inorganic carbon over time. This involved measuring the excess of CO_2_ formed in the vessels containing IL relative to blank containers that monitored the CO_2_ endogenous production. The extent of biodegradation was calculated as a percentage of the theoretical maximum production of inorganic carbon based on the initial concentration of surfactant. In accordance with the method’s pass level of 60%, a chemical with a higher proportion of biodegradation can be regarded as readily biodegradable.

### 3.9. Aquatic Toxicity Test

The *V. fisheri* bioluminescence assay [35] was used to assess the aquatic toxicity of ester-functionalized choline-based ILs. The toxicity of the tested cationic amphiphiles was assessed using luminescent bacteria, where exposure to the compounds resulted in a decrease in light emission proportional to the sample’s toxicity. We determined the concentration causing a 50% reduction in bacterial light emission (EC50) through regression analysis. The reported toxicity data are based on a 30-min exposure of the bacteria to the ionic liquid solution at 15 °C.

## 4. Conclusions

A series of cleavable ionic liquids containing a labile ester group between the hydrocarbon chain and the polar choline group have been synthesized. The presence of an ester group in the side alkyl chain has been demonstrated to significantly reduce the thermal stability of the compound in comparison with non-functionalised choline-based ionic liquids. This observation is consistent with the findings of previous research conducted on other types of ionic liquids. These compounds exhibited higher surface activity and formed aggregates in aqueous solution at lower concentrations than their corresponding non-functionalized choline-based ionic liquids. The aquatic toxicity of CnECholBr was observed to increase with increasing hydrophobicity; however, there is some stabilization for the longer homologues due to their reduced availability in the aqueous medium. Furthermore, the biodegradation rates of CnECholBr were found to be enhanced in comparison to non-functionalized counterparts. However, antimicrobial and antibiofilm activity was found to be significantly lower in comparison to non-functionalized choline-based ILs. Among the compounds evaluated, C_12_ECholBr was identified as the most potent antimicrobial and antibiofilm agent. This research proposes a promising approach to balance antimicrobial efficacy with improved biodegradability in choline-based ionic liquids, potentially leading to more environmentally friendly antimicrobial agents.

## Figures and Tables

**Table 1:** Thermal decomposition temperatures (T_onset_) of ester-functionalized and non-functionalized cholinium-, imidazolium-, and pyridinium-based ionic liquids (ILs).

Ionic Liquid	T_onset1_ (°C)	T_onset2_ (°C)
**Ester-functionalized cholinium ILs (C_10_-C_14_)**	156–164	230–236
Cholinium ILs (C_10_-C_16_)	238–241 [21]	
**Ester-functionalized imidazolium ILs (C_6_-C_14_)**	210–228 [24]	
Imidazolium ILs (C_10_-C_14_)	282–288 [9]	
**Ester-functionalized pyridinium ILs (C_6_-C_14_)**	158–162 [24]	
Pyridinium ILs (C_12_)	232 [9]	

**Table 2:** CMC of ester-functionalized choline-based ILs in aqueous solution.

CnECholBr	CMC ^a^ (mM)	CMC ^b^ (mM)
C_10_ECholBr	11.2 ± 0.8	9.0 ± 0.5
C_12_ECholBr	4.2 ± 0.5	2.9 ± 0.1
C_14_ECholBr	0.7 ± 0.1	1.3 ± 0.1

^a^ conductivity, ^b^ fluorescence.

**Table 3:** MIC values for ester-functionalized choline based ILs against bacteria and yeasts.

		MIC (µg/mL)		
	Microorganism	C_10_ECholBr	C_12_ECholBr	C_14_ECholBr
***Gram-positive bacteria***	*Micrococcus luteus*	>256	16	8
	*Staphylococcus epidermidis*	>256	64	64
	*Staphylococcus aureus*	>256	64	128
	*MRSA*	>256	64	>256
	*Listeria monocytogenes*	>256	>256	>256
***Gram-negative bacteria***	*Escherichia coli*	>256	>256	>256
	*Acinetobacter baumannii*	>256	>256	>256
	*Klebsiella aerogenes*	>256	>256	>256
	*Pseudomonas aeruginosa*	>256	>256	>256
	*Salmonella enterica*	>256	>256	>256
***Yeast***	*Candida albicans*	>256	64	>256
	*Candida tropicalis*	>256	>256	>256
	*Candida parasilopsis*	>256	>256	>256
	*Candida glabrata*	>256	32	>256

**Table 4:** Hemolytic activity of ester-functionalized cholinium-based ILs.

Ester-Functionalized Cholinium-Based IL	HC_50_ (µM)
C_10_ECholBr	>2500
C_12_ECholBr	232 ± 2
C_14_ECholBr	56 ± 1

**Table 5:** Percentage biodegradation of ester-functionalized cholinium-based ILs and the reference substance NaBz (95% confidence limits over 28 days bases on four replicates).

Compound	Biodegradation (%)			
	7 Days	14 Days	21 Days	28 Days
NaBz	80	91	93	98 ± 5.1
C_10_ECholBr	10	19	40	64 ± 2.9
C_12_ECholBr	26	28	46	69 ± 5.5
C_14_ECholBr	29	34	57	61 ± 3.0

**Table 6:** Toxicity results of CnECholBr in the *Vibrio fisheri* luminescence inhibition assay expressed as EC_50_ concentration with their 95% confidence intervals.

Ionic Liquid	*V. fisheri*, EC_50_ (95% CI) (mg/L)
C_10_ECholBr	1.7 (1.4–2.2)
C_12_ECholBr	0.27 (0.12–0.61)
C_14_ECholBr	0.44 (0.14–1.37)

## Data Availability

The data presented in this study are available within this article. The analyzed data sets generated during the present study are available from the corresponding author upon reasonable request.

## Supplementary Materials

The following supporting information can be downloaded at: https://www.mdpi.com/article/10.3390/molecules30061280/s1, Pages S1–S5: Synthesis procedure and characterization data; Figures S1–S6: ^1^H and ^13^C-NMR spectra of CnECholBr; Figures S7–S9: TGA decomposition curves for CnECholBr; Figures S10–S11: Specific conductivity vs. IL concentration for CnECholBr.
