# Supplementary material for: Self-Assembly, Antimicrobial Properties and Biodegradability of Ester-Functionalized Choline-Based Surface-Active Ionic Liquids
Source: Molecules. 2025 Mar 12;30(6):1280. doi: 10.3390/molecules30061280 (PMC11946258; doi:10.3390/molecules30061280)
Supplement: Supplementary file 1 [file molecules-30-01280-s001.zip › molecules-3500396-supplementary.pdf]

Supplementary Materials

Self-assembly, antimicrobial properties and biodegradability of ester-functionalised cholinium-based surface-active ionic liquids

M. Teresa Garcia*, Elena Bautista, Lourdes Pérez, Sergio Vázquez

Department of Surfactants and Nanobiotechnology, Institute of Advanced Chemistry of Catalonia (IQAC-CSIC), Jordi Girona 18-26, 08034, Barcelona, Spain

*Corresponding autor. E-mail address: teresa.garcia@iqac.csic.es

SYNTHESIS SECTION

Materials

2-dimethylaminoethanol (≥99.5%), 2-bromoacetyl bromide, 1-decanol, 1-dodecanol, 1-tetradecanol were purchased from Sigma–Aldrich (Darmstadt, Germany). All other chemicals used were of reagent grade and used without further purification. Deuterated methanol was purchased from Euriso-Top. Water from a Milli-Q Millipore system with electrical resistivity of 18.2 MΩ/cm was used was used to prepare all aqueous solutions.

Synthesis of ester-functionalized cholinium-based ionic liquids

The synthesis of 2-(alkyloxy)-*N*-(2-hydroxyethyl)-*N*,*N*-dimethyl-2-oxoethan-1-aminium bromides(CnECholBr) was performed in two steps according to standard methodology (Garcia et al., 2013; Morrissey et al., 2009; Tehrani-Bagha & Holmberg, 2010). First, commercially available alcohols were reacted with bromoacetyl bromide to obtain the desired alkylating agents. The subsequent alkylation of 2-dimethylaminoethanol with these alkylating agents allows to obtain the corresponding ester-functionalised cholinum-based bromide.

General preparation of alkylbromoacetates

Bromoacetyl bromide (50 mmol) in dichloromethane (10 mL) was added dropwise to a stirred solution of the appropriate primary alcohol (38 mmol) in dichloromethane (40 mL). The reaction mixture was stirred at room temperature overnight. Excess bromoacetyl bromide was removed by washing with a 5% solution of sodium hydrogen carbonate (3 x 70 mL) and the organic phase was dried over magnesium sulfate, filtered and rotary-evaporated. The corresponding alkyl bromoacetate was isolated.

General procedure for the synthesis of 2-(alkyloxy)-N-(2-hydroxyethyl)-N,N-dimethyl-2-oxoethan-1-aminium bromides [CnECholBr]

To a stirred solution of 2-dimethylethanolamine (30 mmol) in toluene (20 mL) at room temperature under nitrogen atmosphere was added dropwise the appropriate alkyl-2-bromoacetate (36 mmol). The reaction mixture was stirred vigorously at room temperature for 1h, then at 65 ºC overnight. The completion of the reaction was

marked by the separation of a solid from the initially obtained clear and homogeneous mixture of the starting materials in toluene. The product was isolated by decanting the toluene layer or by filtration under vacuum through a porous plate. Subsequently, the IL was washed with diethyl ether (50-70 mL). The IL was finally dried under reduced pressure in order to get rid of all the volatile organic compounds.

Analytical data and spectra assignments.
^{1}H and ^{13}C NMR spectra of the purified products were recorded in D_2O or CD_3OD (Euriso-Top, Cambridge, UK) on a Varian spectrometer (Palo Alto, California, US) at 400 MHz (1H) and 101 MHz (13C). HRMS identification of the compounds was performed on an Acquity UPLC System and a LCT Premier™ XE Benchtop orthogonal acceleration TOF (Waters Corporation, Milford, Massachusetts, US) with an electrospray ionization source. Data were processed using MassLynx software (v 4.2).

2-(decyloxy)-N-(2-hydroxyethyl)-N,N-dimethyl-2-oxoethan-1-aminium bromide. [C_{10}ECholBr] was obtained as a white solid. Yield: 68%. ^{1}H NMR (400 MHz, MeOD) δ 4.47 (d, *J* = 7.3 2H), 4.24 (t, *J* = 6.7 Hz, 2H), 4.03 – 3.93 (m, 2H), 3.81 – 3.74 (m, 2H), 3.40 (d, *J* = 9.8 Hz, 6H), 1.68 (dt, *J* = 8.1, 6.4 Hz, 2H), 1.43 – 1.29 (m, 7H), 1.29 (s, 8H), 0.94 – 0.86 (m, 2H). ^{13}C NMR (101 MHz, MeOD) δ 166.26, 67.42, 67.25, 63.01, 56.87, 54.20, 53.62, 30.76, 30.73, 30.69, 30.63, 30.46, 29.47, 26.86, 23.72, 14.43. HRMS (ESI) m/z 288.2542 ($[M]^+$; calculated for $[C_{16}H_{34}NO_3]^+$: 288.2534).

2-(dodecyloxy)-N-(2-hydroxyethyl)-N,N-dimethyl-2-oxoethan-1-aminium bromide. [C_{12}ECholBr] was obtained as a white solid. Yield: 72%. ^{1}H NMR (400 MHz, MeOD) δ 4.46 (s, 2H), 4.24 (t, *J* = 6.7 Hz, 2H), 4.03 – 3.94 (m, 2H), 3.77 (dq, *J* = 5.1, 2.5 Hz, 2H), 3.39 (s, 6H), 1.68 (dt, *J* = 8.3, 6.6 Hz, 2H), 1.33 – 1.26 (m, 7H), 1.29 (s, 8H), 0.93 – 0.83 (m, 2H). ^{13}C NMR (101 MHz, MeOD) δ 165.6, 66.8, 66.6, 62.4, 56.2, 53.0, 33.0, 32.4, 30.1, 30.1, 30.1, 30.0, 29.8, 29.7, 28.8, 26.2, 23.1, 13.8. HRMS (ESI) m/z 316.2854 ($[M]^+$; calculated for $[C_{18}H_{38}NO_3]^+$: 288.2534).

2-(tetradecyloxy)-N-(2-hydroxyethyl)-N,N-dimethyl-2-oxoethan-1-aminium bromide. [C_{14}ECholBr] was obtained as a white solid. Yield: 77%. ^{1}H NMR (400 MHz, MeOD) δ 4.46 (s, 2H), 4.24 (t, *J* = 6.6 Hz, 2H), 4.03 – 3.92 (m, 2H), 3.77 (dq, *J* = 5.3, 2.6 Hz, 2H), 3.39 (s, 6H), 1.68 (dt, *J* = 8.4, 6.6 Hz, 2H), 1.45 – 1.30 (m, 7H), 1.29 (s, 10H), 0.97 – 0.86 (m, 2H). ^{13}C NMR (101 MHz, MeOD) δ 166.3, 67.4, 67.2, 63.0, 56.9, 53.6, 33.1, 30.8, 30.8, 30.7, 30.7, 30.6, 30.6, 30.5, 30.3, 29.5, 27.0, 26.9, 23.7, 14.4. HRMS (ESI) m/z 344.3169 ($[M]^+$; calculated for $[C_{20}H_{42}NO_3]^+$: 344.3160).

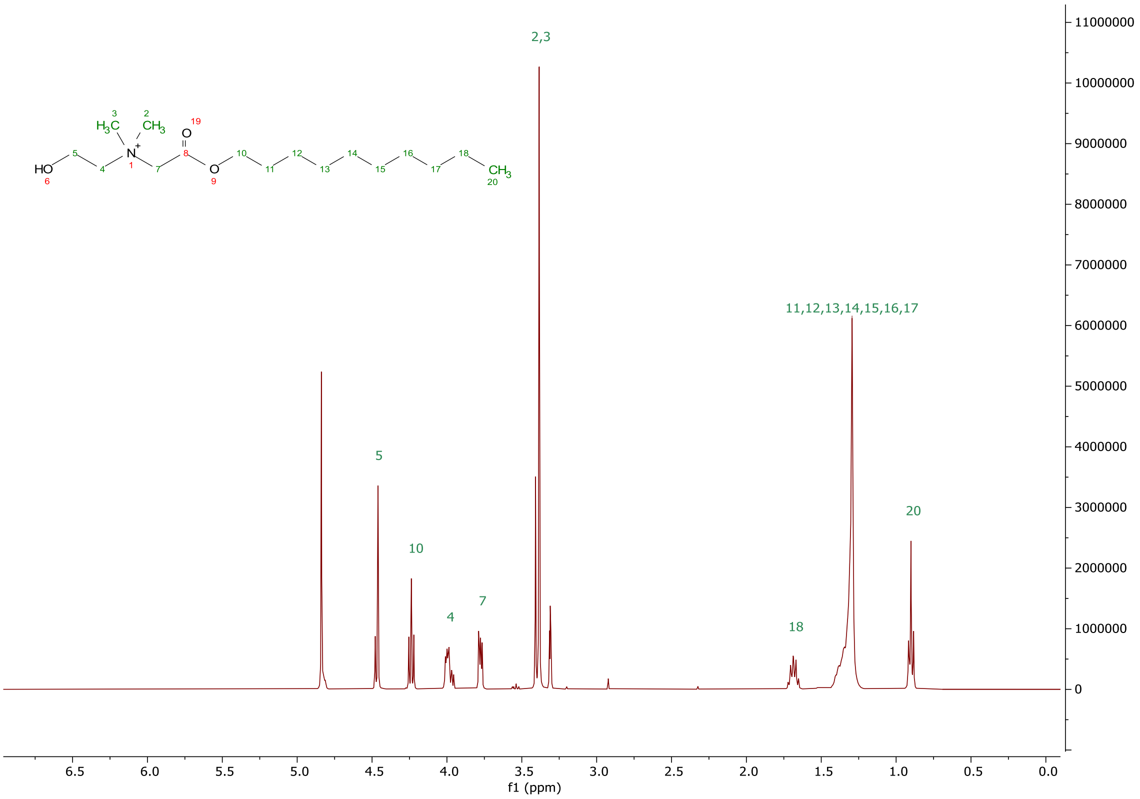

Figure S1: ^{1}H-NMR spectrum of C_{10}ECholBr in CD_3OD

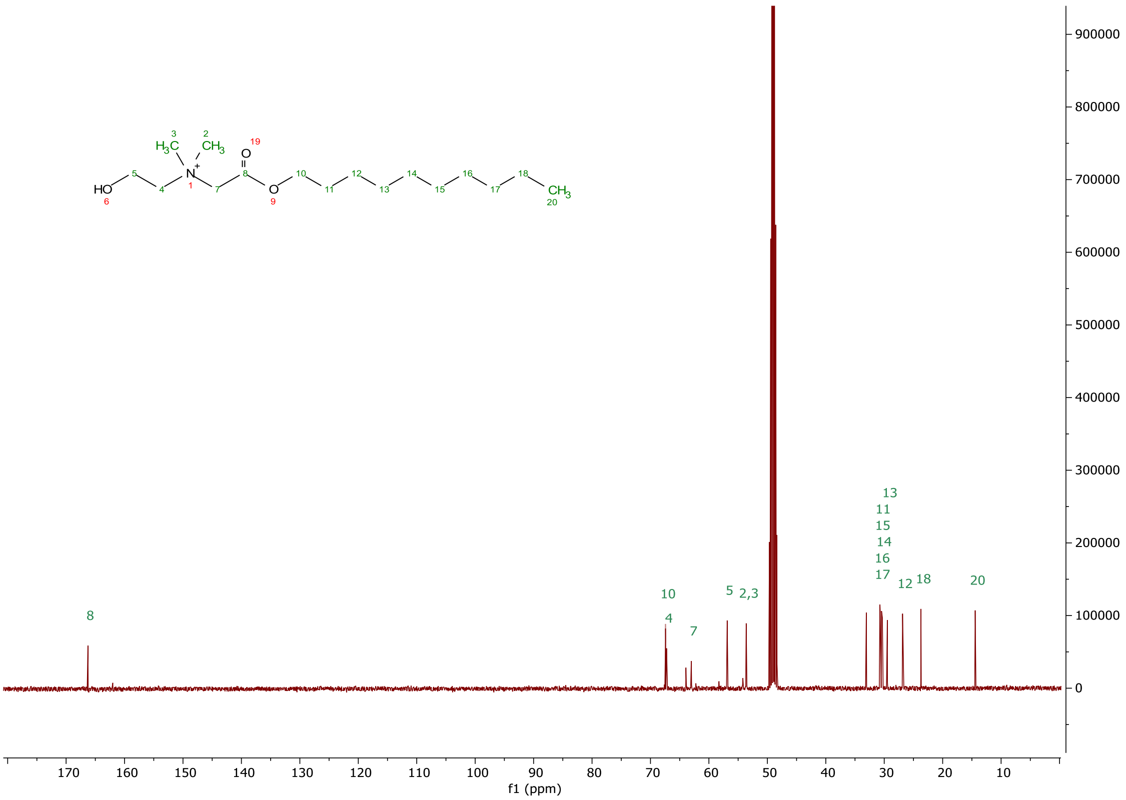

Figure S2: ^{13}C-NMR spectrum of C_{10}ECholBr in CD_3OD

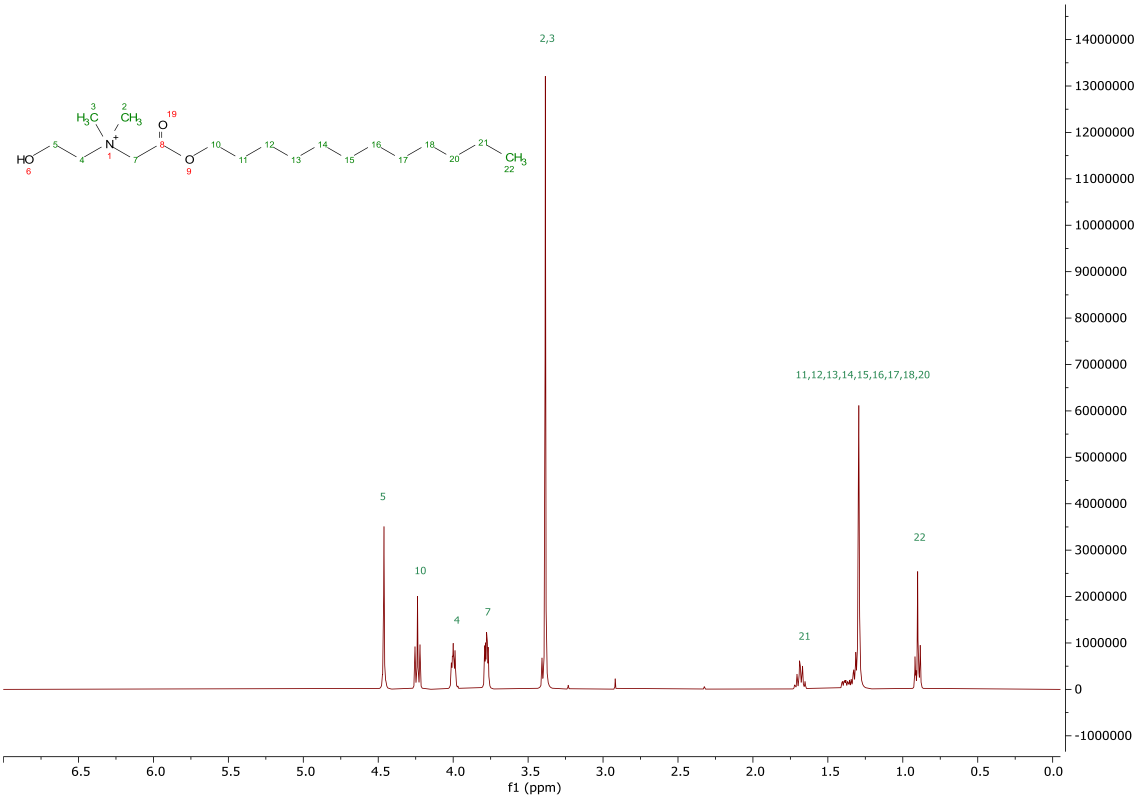

Figure S3: ^{1}H-NMR spectrum of C_{12}ECholBr in CD_3OD

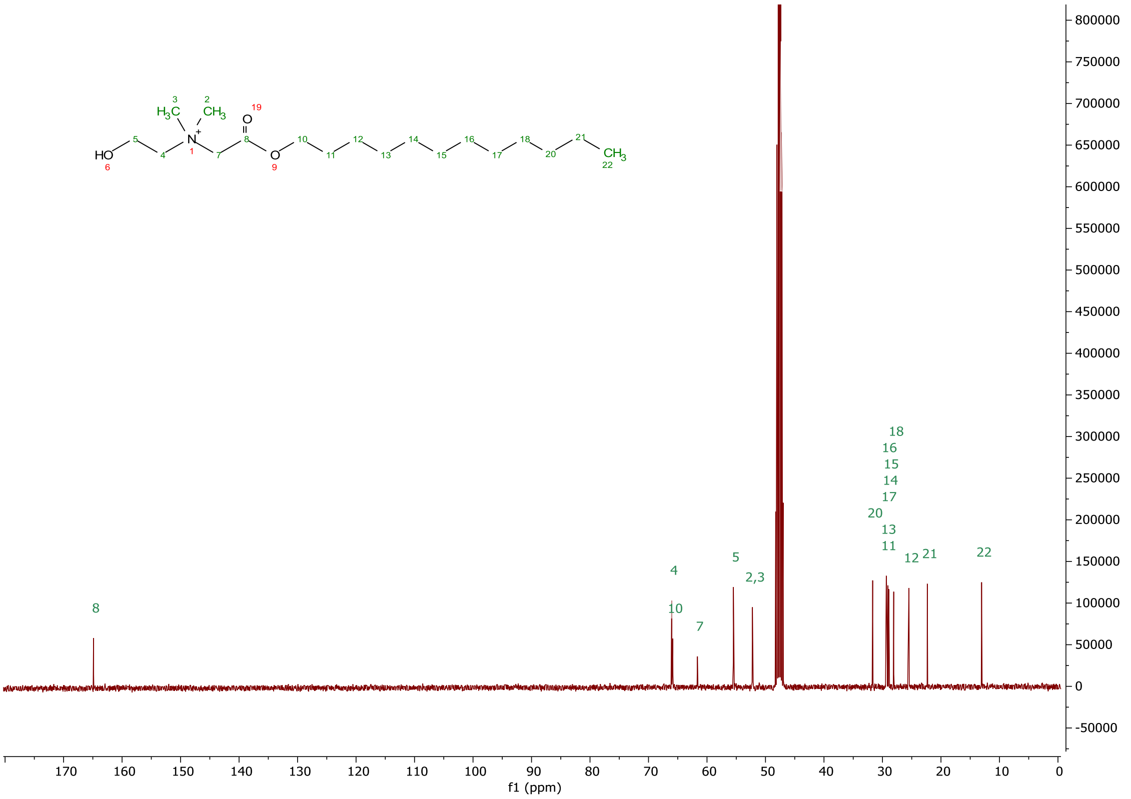

Figure S4: 13C-NMR spectrum of C_{12}ECholBr in CD_3OD

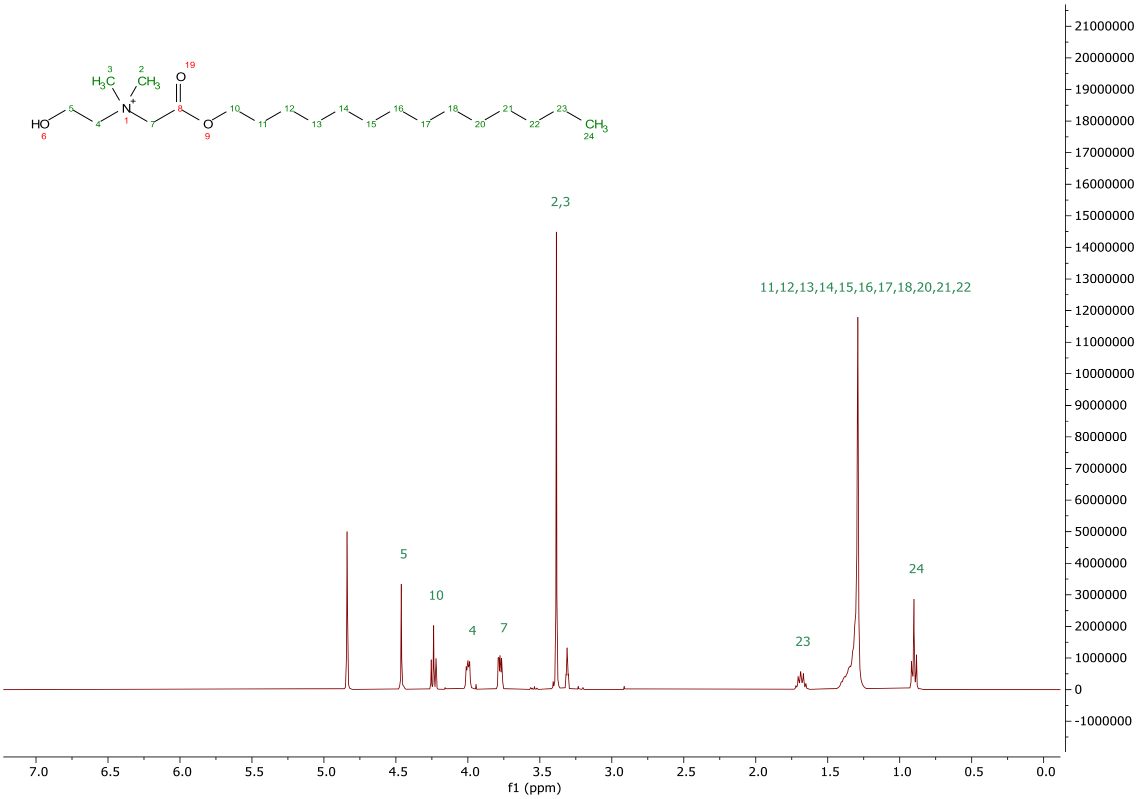

Figure S5: ^{1}H-NMR spectrum of C_{14}NicBr in CD_3OD

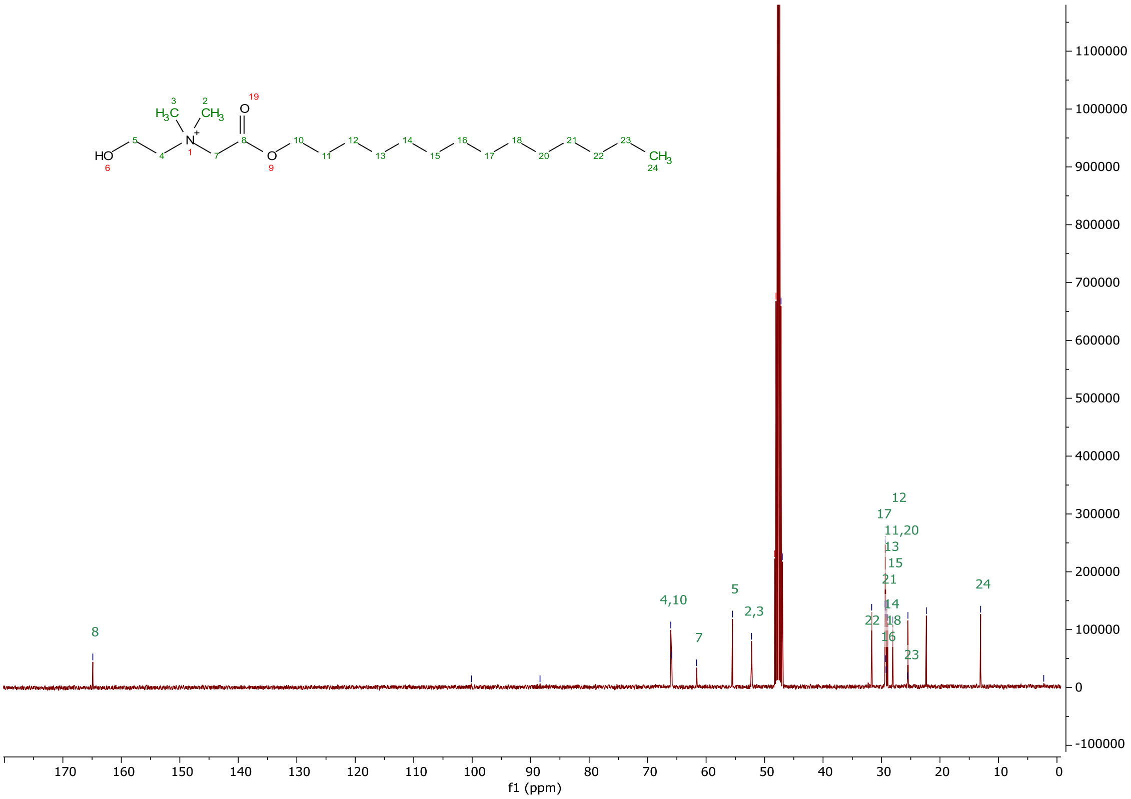

Figure S6: ^{13}C-NMR spectrum of C_{14}NicBr in CD_3OD

THERMAL STABILITY

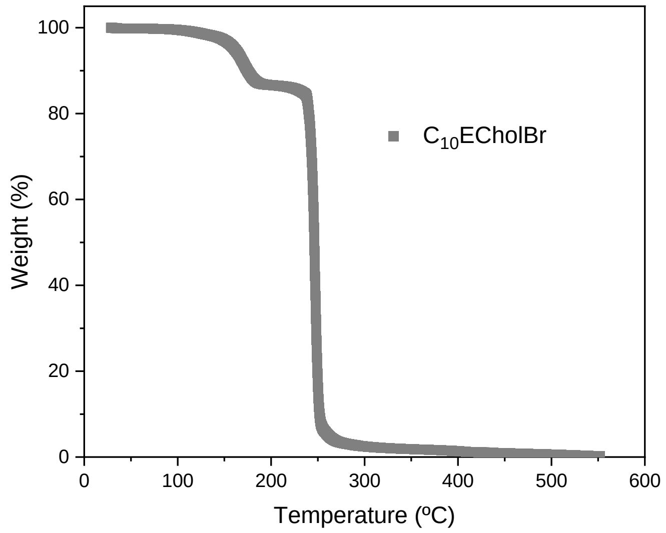

Figure S7. Decomposition curve determined by TGA for C_{10}ECholBr

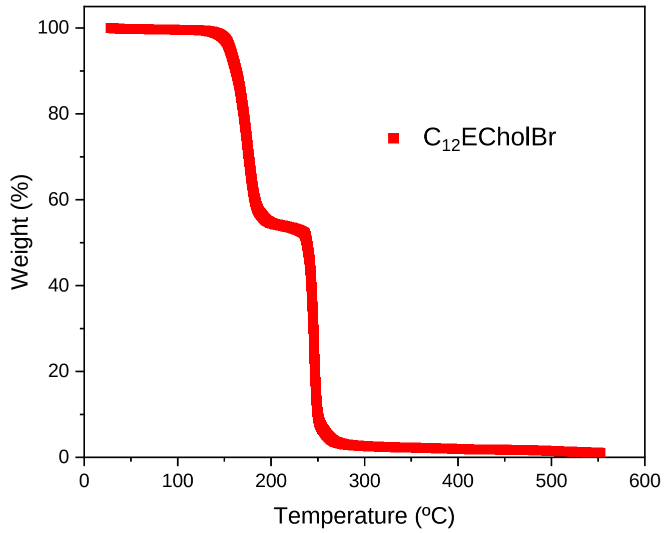

Figure S8. Decomposition curve determined by TGA for C_{12}ECholBr

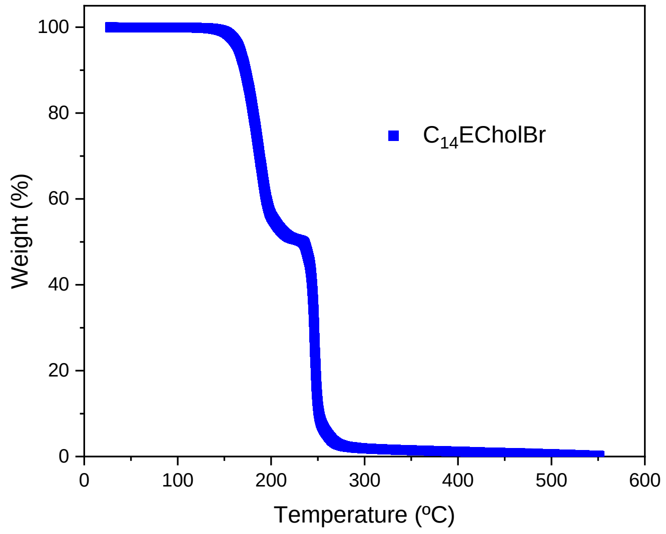

Figure S9. Decomposition curve determined by TGA for C_{14}ECholBr

CONDUCTIVITY MEASUREMENTS

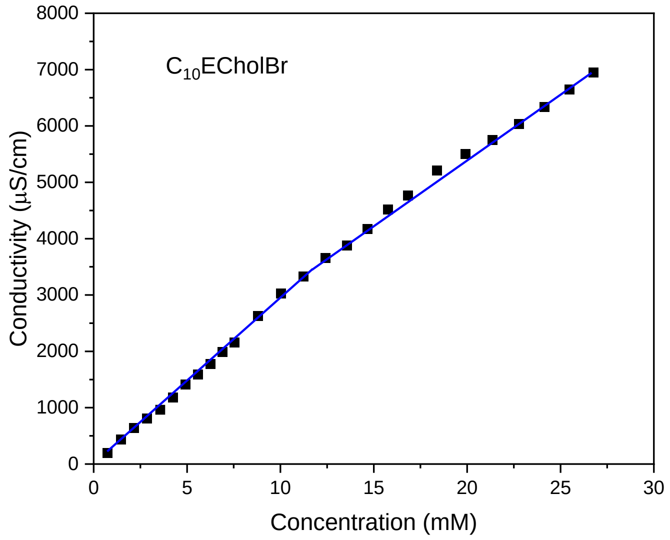

Figure S10. Specific conductivity versus ionic liquid concentration in water at 25 ºC for C_{10}ECholBr.

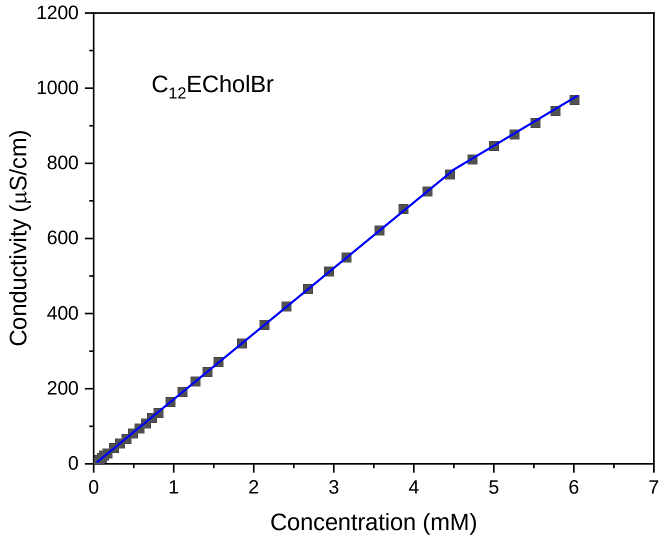

Figure S11. Specific conductivity versus ionic liquid concentration in water at 25 ºC for C_{12}ECholBr.
